# Retraction Note: Ginsenoside Rg3 alleviates septic liver injury by regulating the lncRNA TUG1/miR-200c-3p/SIRT1 axis

**DOI:** 10.1186/s12950-025-00435-z

**Published:** 2025-02-18

**Authors:** Pan Wu, Xiao Yu, Yue Peng, Qian-Lu Wang, Long-Tian Deng, Wei Xing

**Affiliations:** 1 Hematology Department, Hunan Children’s Hospital, Changsha, 410007 Hunan Province P.R. China; 2 Department of Nursing, The Third Xiangya Hospital of Central South University, Changsha, 410013 Hunan Province P.R. China; 3 Department of Critical Care Medicine, The Third Xiangya Hospital of Central South University, No.138, Tongzipo Road, Changsha, 410013 Hunan Province P.R. China

**Retraction Note: Journal of Inflammation (2021) 18:31**

10.1186/s12950-021-00296-2

The Editor-in-Chief has retracted the article. Concerns were raised regarding a similar image in Fig. 5D. The authors sent partial data upon request, but this did not address the concerns raised, and further concerns regarding data in Fig. 1 were found. The authors were also unable to provide the ethics approval documents upon request. Therefore, the Editor has lost the confidence in the data and results presented here.

Author Wei Xing has stated on behalf of authors Pan Wu, Xiao Yu, Yue Peng, Qian-Lu Wang, and Long-Tian Deng that all authors agree with this retraction.

